# Endoplasmic reticulum stress mediates resistance to BCL-2 inhibitor in uveal melanoma cells

**DOI:** 10.1038/s41420-020-0259-2

**Published:** 2020-04-17

**Authors:** Lara Bellini, Thomas Strub, Nadia Habel, Charlotte Pandiani, Sandrine Marchetti, Arnaud Martel, Stéphanie Baillif, Béatrice Bailly-Maitre, Philippe Gual, Robert Ballotti, Corine Bertolotto

**Affiliations:** 1 Université Nice Côte d’Azur, Inserm, C3M Nice, France; 2 INSERM, U1065, Biology and pathologies of melanocytes, team 1. Equipe labellisée Ligue 2020, Nice, France; 3 INSERM, U1065, Metabolism, cancer and immune response, team 3, Nice, France; 4 CHU NICE, Département d’Ophtalmologie, Nice, France; 5 INSERM, U1065, Chronic liver diseases associated with obesity and alcohol, team8, Nice, France

**Keywords:** Eye cancer, Cell death

## Abstract

To address unmet clinical need for uveal melanomas, we assessed the effects of BH3-mimetic molecules, the ABT family, known to exert pro-apoptotic activities in cancer cells. Our results uncovered that ABT-263 (Navitoclax), a potent and orally bioavailable BCL-2 family inhibitor, induced antiproliferative effects in metastatic human uveal melanoma cells through cell cycle arrest at the G0/G1 phase, loss of mitochondrial membrane potential, and subsequently apoptotic cell death monitored by caspase activation and poly-ADP ribose polymerase cleavage. ABT-263-mediated reduction in tumor growth was also observed in vivo. We observed in some cells that ABT-263 treatment mounted a pro-survival response through activation of the ER stress signaling pathway. Blocking the PERK signaling pathway increased the pro-apoptotic ABT-263 effect. We thus uncovered a resistance mechanism in uveal melanoma cells mediated by activation of endoplasmic reticulum stress pathway. Therefore, our study identifies ABT-263 as a valid therapeutic option for patients suffering from uveal melanoma.

## Introduction

Uveal melanoma is the most common primary intraocular malignancy in adult population^1,2^. Despite enucleation or radiotherapy of the primary lesion, metastases develop in 50% of patients, mainly to the liver. These metastases are remarkably refractory to conventional chemotherapies, immunotherapy with checkpoint inhibitors and external radiotherapy^3,4^. The median survival of patients who develop liver metastasis is reported to be 4 to 15 months, and the one-year survival rate is estimated to be 10–15%^4^. This highlights an urgent need for an efficient treatment.

Defective apoptosis, which contributes to sustained cell survival, is a major causative factor in the development and progression of cancer. The ability of a cell to undergo apoptosis is governed by members of the BCL-2 protein family that are grouped into three sub-families based on the number of BH (BCL-2 Homology) domains they share (BH1-4). They can be anti-apoptotic (e.g., BCL-2, BCL-XL, MCL-1) or pro-apoptotic (e.g., BAX, BID, BIM, NOXA)^5^, among which some of them only contain the BH3 domain^6^.

BCL-2 can exert its anti-apoptotic function by sequestering the activator BH3-only proteins or through direct interaction with apoptosis-activating factors such as BAX (BCL-2 associated-X-protein) and BAK (BCL-2 homologous antagonist/killer), thereby modulating mitochondrial cytochrome c release. The release of cytochrome c subsequently leads to caspase 9 activation and to apoptosome formation, which activates the other caspases (caspases 3–7), ending in cell apoptosis.

Overexpression of the pro-survival BCL-2 family members is commonly associated with cancer^7^. Such deregulations can be exploited by chemotherapeutic strategies, such as the BH3-mimetic drugs, which inhibit the antiapoptotic proteins by occupying their BH3-binding groove, to counteract the apoptotic blocks, and halt tumor progression^8,9^. Several BH3 mimetics, including ABT-737, ABT-263 (Navitoclax) and ABT-199 (Venetoclax) have been developed as cancer therapeutics^10^. ABT-263 (Navitoclax), an orally available derivative of ABT-737, was tested as a single agent in phase I/II for the treatment of different solid and haematological malignancies, yet side effects such as thrombocytopenia have been reported^9,11,12^. ABT-199 is an oral second-generation BH3 mimetic that inhibits BCL-2, with much less activity against BCL-xL. It is the first BH3 mimetic drug approved by the US Food and Drug Administration for the treatment of some leukemias and lymphomas^13^.

Alternatively, BCL-2 family members are contained in other multiprotein complexes at the endoplasmic reticulum (ER) that are involved in the control of diverse cellular processes including calcium homeostasis and autophagy to regulate the switch between adaptive and proapoptotic phases under stress. Increasing evidence indicates that a functional activity of BCL-2 on ER protects mitochondria under diverse circumstances^14^.

In primary uveal melanoma, expression of BCL-2 is significantly higher compared to normal ocular structures, or choroidal melanocytes^5,15^, suggesting that it may be involved in the development and progression of these lesions. However, effects of ABT drugs have not been assessed.

In this study, we showed in vitro and in vivo that ABT-263 has antiproliferative and proapoptotic activities in uveal melanoma cells derived from primary tumors and metastases. We demonstrated that the effect of ABT-263 in some cells is accompanied with the activation of the ER stress response pathway that exerted a cytoprotective effect. Blocking ER stress enhanced ABT-263 efficacy.

## Results

### ABT-263 triggers apoptotic cell death of uveal melanoma cells

**Fig. 1:**
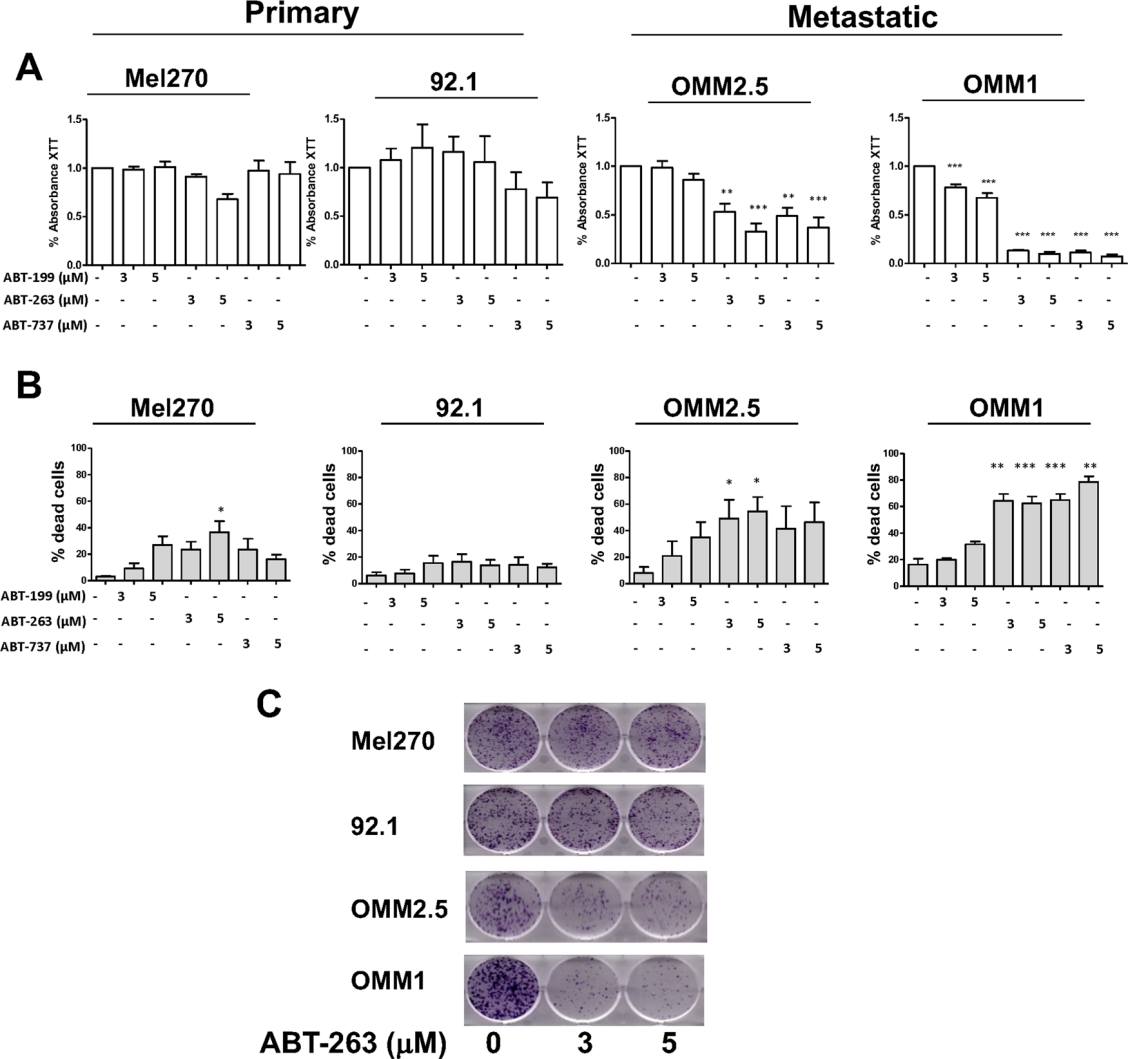
Effect of ABT drugs on uveal melanoma cells. **a** Viability test (XTT) after exposure of a panel of uveal melanoma cell lines to BH3 mimetics drugs for 48 h. Results are represented as means + SD of a minimum of three independent experiments, **P-value < 0.01, ***P-value < 0.001. **b** FACS analyses of Annexin V/DAPI double staining in a panel of uveal melanoma cells indicate dead (early, late apoptosis and necrosis in light grey) cells after exposure to BH3 mimetics drugs for 48 h. Results are represented as means +SD of minimum three independent experiments, *P-value < 0.05, **P-value < 0.01, ***P-value < 0.001. **c** Colony formation assay of primary and metastatic uveal melanoma cells exposed to ABT-263 3 μM and 5 μM. Colonies were stained with crystal violet after 7 days (representative micrographs are shown).

We first conducted experiments to assess the effect of three ABT drugs, ABT-199, ABT-737 and ABT-263 on uveal melanoma cell proliferation using four human uveal melanoma cell lines. Mel270^16^, 92.1^17^ and OMM2.5^16^ were not sensitive to ABT-199, and OMM1^18^ showed a moderate proliferation inhibition (about 15%) (Fig. 1a). Whereas Mel270 and 92.1 were also highly resistant to ABT-737 and ABT-263, both drugs strongly reduced proliferation of OMM1 and OMM2.5 cells. Accordingly, cell death was higher in OMM1 and OMM2.5 cells compared to Mel270 and 92.1 cells (Fig. 1b). Thus, ABT-263 and the related ABT-737 gave similar and superior results to ABT-199. Since ABT-263 is used in clinical trials, we decided to pursue with ABT-263 for subsequent studies.

In agreement with the previous observations, in a colony formation assay, the OMM1 and OMM2.5 cell lines were more sensitive than Mel270 and 92.1 cell lines to the long-term growth inhibitory activity of ABT-263 (Fig. 1c).

Western blot indicated that the effect of ABT-263 was associated with cell cycle arrest in Mel270 cells, as shown by a reduced CDK4 expression and an increase in the cell cycle inhibitors p27 and p21 (Supplementary Fig. 1).

**Fig. 2:**
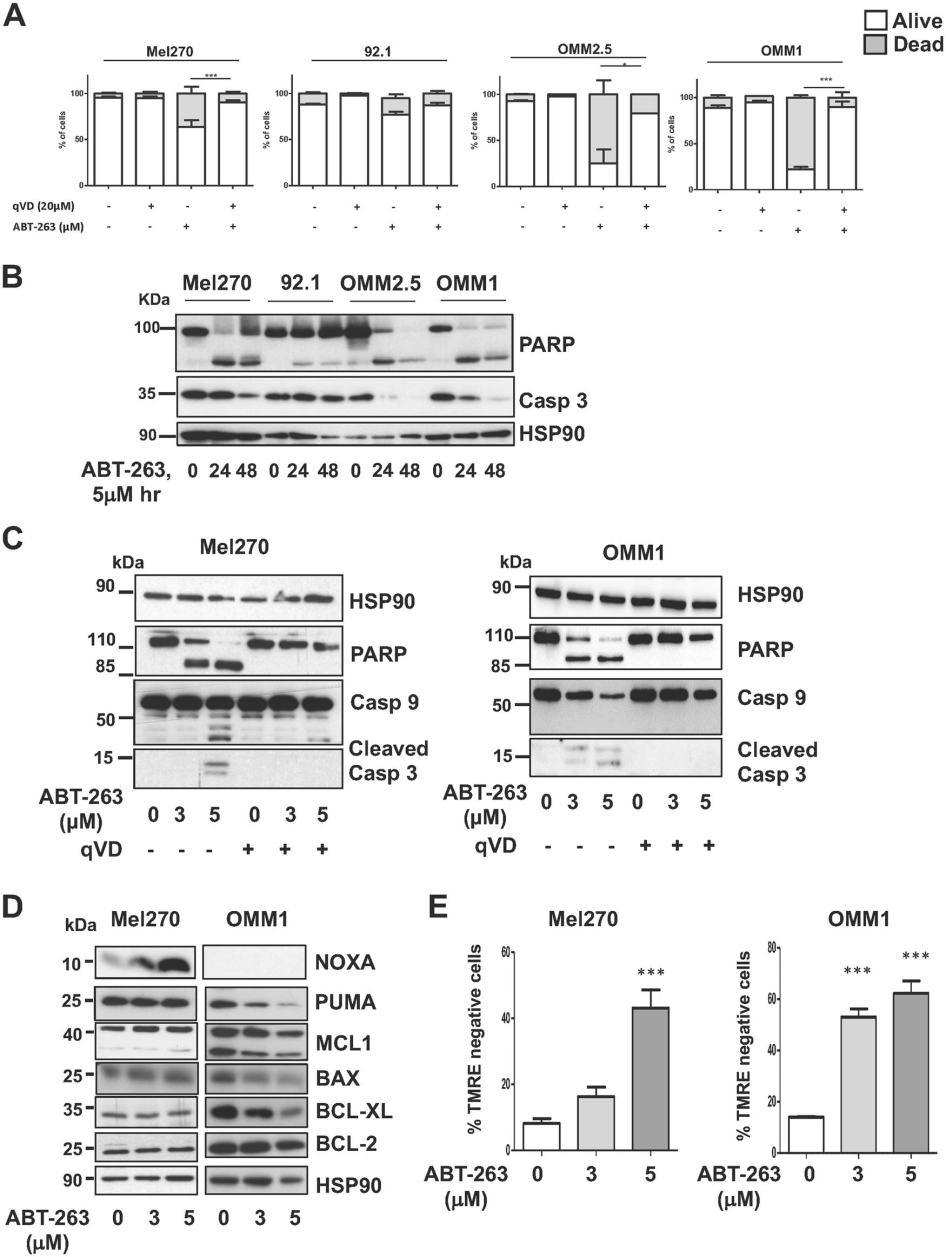
ABT-263 induces apoptosis of uveal melanoma cells. **a** FACS analyses of Annexin V/DAPI double staining in a panel of uveal melanoma cells indicate alive (white) or dead (early, late apoptosis and necrosis in light grey) cells after exposure to ABT-263 5 μM for 48 h in the absence or presence of qVD-OPh 20 μM. Results are represented as means + SD of three independent experiments, *P-value < 0.05, ***P-value < 0.001. **b** Western blot analysis of PARP and caspase 3 in Mel270, 92.1, OMM2.5 and OMM1 uveal melanoma cells exposed to ABT-263 5 μM for 24 h and 48 h. Detection of HSP90 serves as a loading control. Representative immunoblots are shown. **c** Western blot analysis of PARP, caspase 9 and caspase 3 human primary Mel270 and metastatic OMM1 uveal melanoma cells exposed to ABT-263 3 μM or 5 μM for 48 h in the absence or presence of qVD-OPh 20 μM. Detection of HSP90 serves as a loading control. Representative immunoblots are shown. **d** Western blot analysis of pro- and anti-apoptotic members of the BCL-2 family proteins in Mel270 and OMM1 uveal melanoma cells that were left untreated or treated with ABT-263 3 μM or 5 μM for 24 h. HSP90 was used as a loading control. **e** Detection of the mitochondrial membrane potential using TMRE staining in Mel270 and OMM1 uveal melanoma cells exposed to ABT-263 3 μM or 5 μM for 48 h. Percent of TMRE negative cells is shown. ***P-value < 0.001.

We next assessed the ability of ABT-263 to induce apoptosis in the cell line panel. ABT-263 cytotoxicity as measured by Annexin V/DAPI staining revealed that OMM1 and OMM2.5 uveal melanoma cells were much more sensitive than Mel270 and 92.1 uveal melanoma cell lines, inducing 80% versus 20–35% cell death, respectively (Fig. 2a). The pan-caspase inhibitor qVD triggered a protective effect in all cell lines. Additionally, ABT-263 induced a time-dependent decrease of the full-length PARP protein, and caspase 3 zymogen (Fig. 2b) in the different cell lines. The disappearance of total poly ADP-ribose polymerase (PARP) and of the zymogenic form of caspase 3 appeared weaker in Mel270 and 92.1 cells compared to OMM1 and OMM2.5 cells.

A dose-dependent cleavage of PARP, caspase 9 and caspase 3 was also observed in all cell lines (Fig. 2c). Cleavage of caspase 3 was detected earlier in the OMM1 metastatic cells than in Mel270 cells. As confirmation that cell death was due to apoptosis, pretreatment with the pan caspase inhibitor qVD prevented both PARP and caspase cleavage (Fig. 2c).

Protein immunoblot revealed that the expression level of the main anti-apoptotic BCL-2 subfamily members (BCL-2, MCL1, BCL-xL) was higher in OMM1 cell line compared to Mel270 cells (Fig. 2d). After 24 h of treatment, the only noticeable change in primary cells was the increase in pro-apoptotic NOXA level (Fig. 2d). In OMM1 cells, but not in Mel270 cells, due to significant cell death, ABT-263 effect was accompanied by a reduced expression of pro-survival (BCL-xL, MCL1), and pro-apoptotic (PUMA, BAX) members (Fig. 2d). BCL-2 exhibited no change in response to ABT-263.

BH3-only proteins control the mitochondrial pathway to apoptosis. We therefore measured the mitochondrial membrane potential by tetramethylrhodamine ethyl ester (TMRE) staining. ABT-263 dose-dependently reduced the mitochondrial membrane potential in both Mel270 and OMM1 cells (Fig. 2e). Interestingly, while qVD treatment did not prevent mitochondrial membrane depolarization in Mel270 cells (Supplementary Fig. 2a), it blocked this process following ABT-263 exposure in OMM1 cells (Supplementary Fig. 2b), suggesting that different mechanisms of cell death induction operate in these the two cell lines.

Altogether, these data demonstrated that ABT-263-induced cell death via apoptosis in uveal melanoma cells. Notably, OMM1 and OMM2.5 melanoma cells appeared more sensitive to ABT-263 effects than Mel270 and 92.1 cells.

### ABT-263 prevents growth of human uveal melanoma tumor xenografts

Experiments were next conducted in vivo to investigate the antineoplastic effect of ABT-263. For this purpose, the Mel270 and OMM1 human uveal melanoma cells were engrafted subcutaneously on the flank of nude mouse, which were subsequently treated with ABT-263 or with its vehicle.

**Fig. 3:**
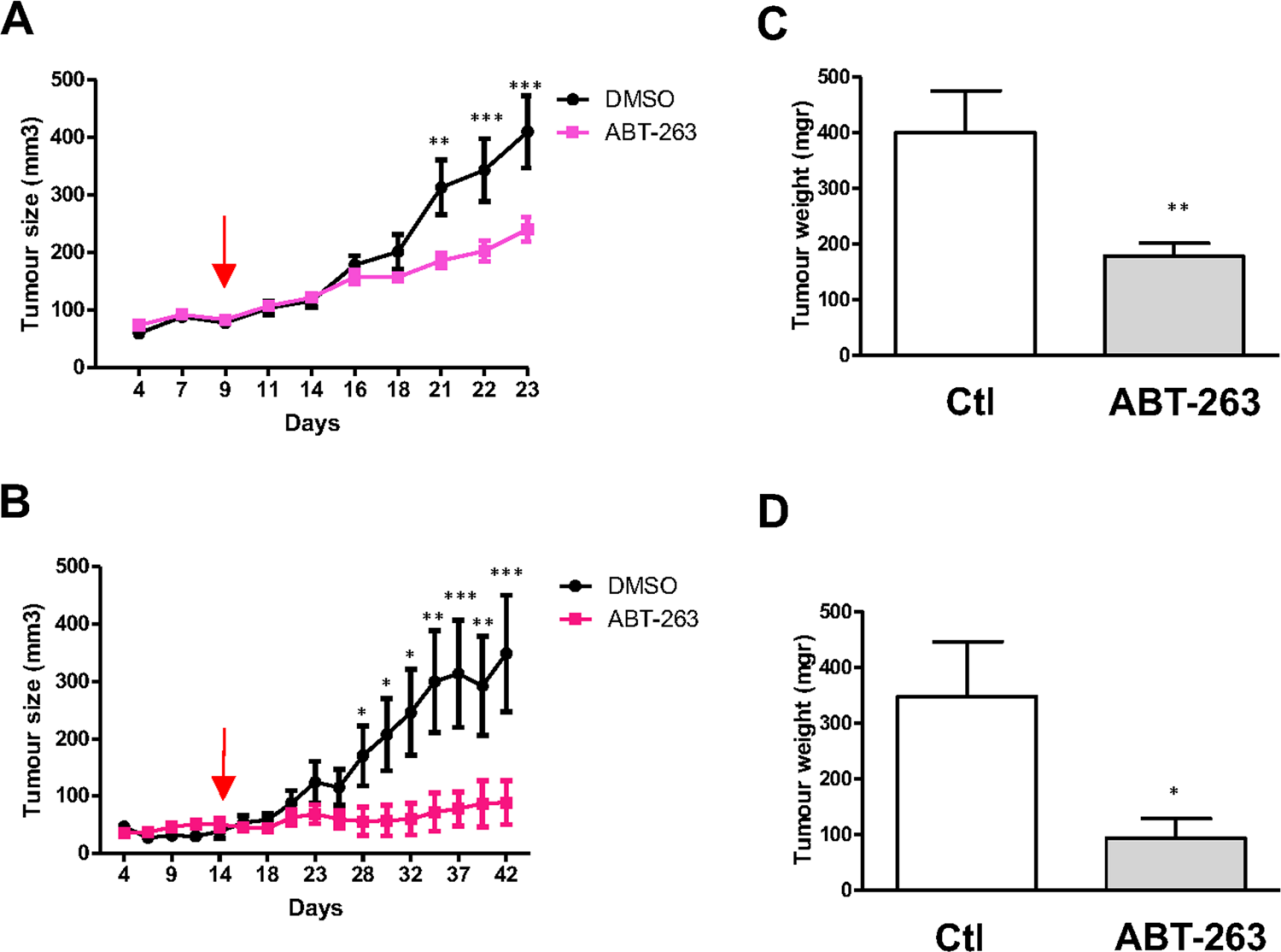
ABT-263 impairs tumor growth in vivo. **a** Mel270 and **b** OMM1 uveal melanoma cells were subcutaneously engrafted into athymic nude mice (*n* = 8 per group). Once the tumours reached 100mm3, mice were treated daily with ABT- 263. The red arrow indicates the start of the treatment. The growth tumor curves were determined by measuring the tumor volume using the equation *V* = (*L* × *W* × *W*)/2, where *V* is tumor volume, *W* is tumor width, *L* is tumor length. Results are presented as mean (±SEM) tumor volumes (mm3). **P-value < 0.01, ***P-value < 0.001 are from 2-way anova test in ABT-263 treated versus vehicle at each point. **c**, **d** The weight of the sub-cutaneous tumor from control (Ct) and ABT-263-treated mice is shown. *P-value < 0.05, **P-value < 0.01.

ABT-263 was found to be partially effective in the group of established Mel270 xenografts (Fig. 3a) whereas it induced marked tumor growth inhibition of metastatic OMM1 xenografts, leading in mice to complete tumor shrinkage (Fig. 3b). Excised tumors in the ABT-263 group weighted significantly less than those in the control group (Fig. 3c, d). There was no significant difference in body weight between mice treated with vehicle or ABT-263, indicating no toxicity issues caused by ABT-263 in vivo. Thus, ABT-263 proved to be highly efficient in killing metastatic uveal melanoma cells and not only prevented tumor growth in vitro but also in vivo.

### ABT-263 induces endoplasmic reticulum (ER) stress

BCL-2 protein family is mainly known for its anti-apoptotic role operating at the mitochondria level, yet it is also recognized for its role in the endoplasmic reticulum (ER)^19^. Since our different cell lines showed variable sensitivity to ABT-263, we decided to investigate whether these cell lines also differed in the effect of ABT-263 on the ER pathway.

ER stress leads to unfolded protein response (UPR) which is sensed by the ER chaperone protein glucose regulated protein 78 (GRP78/also called BIP) and activation of three known ER resident proteins: inositol requiring protein-1 (IRE1), protein kinase RNA-like ER kinase (PERK), and activating transcription factor-6 (ATF6), and then converge to drive the expression of C/EBP homologous protein (CHOP)^20^.

**Fig. 4:**
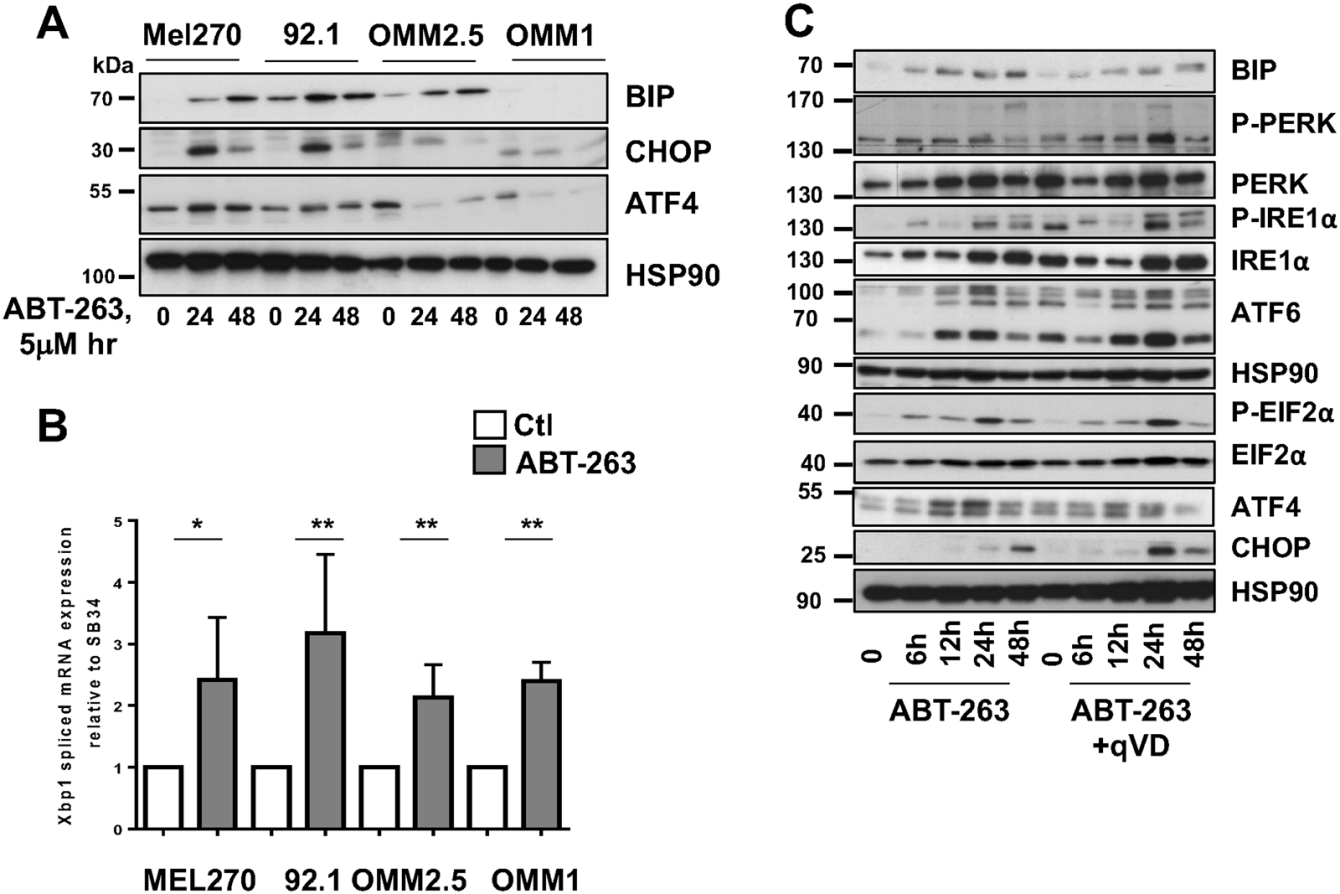
ABT-263 induces ER stress and activation of the unfolded protein response. **a** Western blot of ER stress molecules in uveal melanoma cells treated with ABT-263 5 μM for 24 h and 48 h. HSP90 is used as a loading control. Representative western blots are shown. **b** XBP1 splicing in uveal melanoma cells exposed to ABT-263 5 μM for 15 h. *P-value < 0.05, **P-value < 0.01. **c** Kinetic analysis of ER stress proteins in Mel270 cells treated with ABT-263 5 μM in the absence or presence of qVD-OPh 20 μM. HSP90 is used as a loading control. Representative western blots are shown.

ABT-263-mediated ER stress was analyzed in the cell lines panel using detection of BIP, ATF4 (PERK signaling) and CHOP as surrogates for an ER stress response. In both Mel270 and 92.1 cells, ABT-263 enhanced ATF4 and CHOP as well as BIP level (Fig. 4a). In contrast, induction of ATF4 and CHOP by ABT-263 was hardly detectable in OMM1 and OMM2.5 human uveal melanoma cells, and BIP was not detected in OMM1 cells. We also did not detect PERK and IRE1α phosphorylation (Thy980 and Ser724 respectively) in OMM1 and OMM2.5 cell lines treated with ABT-263 (data not shown).

Of note, in contrast to ABT-263, tunicamycin, another ER stress inducer, was able to stimulate CHOP in OMM1, thus ruling out that the ER stress pathway is deficient in OMM1 and OMM2.5 cell lines (Supplementary Fig. 3). Furthermore, XBP1 splicing was significantly higher following ABT-263 exposure (Fig. 4b).

Kinetics analysis in Mel270 primary uveal melanoma cells revealed that the protein level of BIP was enhanced following treatment with ABT-263 (Fig. 4c). PERK and EIF2α were phosphorylated (Thy980 and Ser51, respectively) and ATF4 increased. We also observed IRE1α phosphorylation and an increase in ATF6 expression and cleavage. There was also a significant upregulation of the very downstream ER stress effector CHOP (Fig. 4c). Treatment with the pan caspase inhibitor qVD did not impact the ABT-263 mediated change in ER molecules. These data indicate that in Mel270 uveal melanoma cells, ABT-263 activated all branches of UPR, and that UPR operated upstream of the apoptotic pathway.

Altogether, our data indicate that ABT-263 elicits activation of the ER stress pathway in Mel270 and 92.1 uveal melanoma cells, while there are signs of defective activation of this pathway in OMM1 and OMM2.5 cells.

### ER stress protects from ABT-263-induced apoptosis

Chemotherapy regimens were previously found to cause ER stress and to activate UPR, which paradoxically is a process that prevents apoptosis of the cancer cells and promotes their survival.

To delineate the role of ER stress pathway activation in ABT-263 effects, we used genetic or pharmacological intervention to target these pathways. Although ATF6 increases XBP1 expression, the spliced form of XBP1 that activates the UPR efficiently is mediated by IRE1α^21^. We were therefore focusing on PERK and IRE1α, which are the two main branches.

**Fig. 5:**
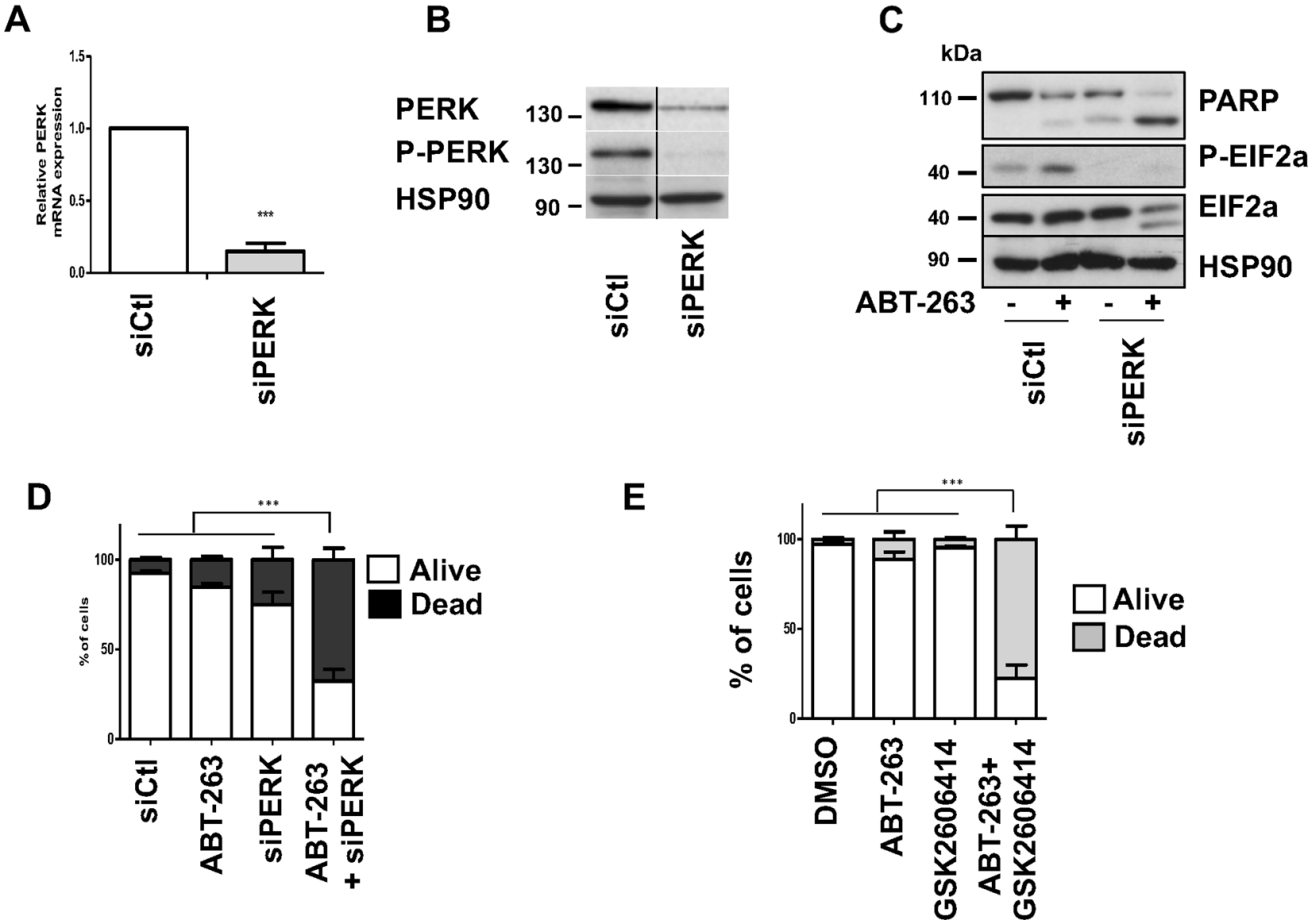
PERK dampens ABT-263 killing activity. **a** Q-PCR analysis of PERK level in 92.1 uveal melanoma cells treated with control (siCtl) or PERK (siPERK) siRNA for 48 h. ***P-value < 0.001. **b** Western blot analysis to PERK and P-PERK in 92.1 cells treated with control (siCtl) or PERP (siPERK) siRNA. HSP90 is used as a loading control. **c** 92.1 uveal melanoma cells treated with control (siCtl) or PERK (siPERK) siRNA for 48 h before being exposed to ABT-263 3 μM for 48 h. Representative western blot to P-EIF2α, total EIF2α and PARP are shown. HSP90 serves as a loading control. **d** 92.1 cells were treated as in **b**. FACS analyses of Annexin V/DAPI double staining in uveal melanoma cells indicate alive (white) or dead (early, late apoptosis and necrosis in light grey) cells. ***P-value < 0.001. **e** 92.1 cells were treated as in **b**. FACS analyses of Annexin V/DAPI double staining in uveal melanoma cells indicate alive (white) or dead (early, late apoptosis and necrosis in light grey) cells. ***P-value < 0.001.

We first analysed siRNA against IRE1α. Whereas it efficiently reduced IRE1α expression in Mel270 and 92.1 cells, it did not enhance cell death mediated by ABT-263 treatment (Supplementary Fig. 4a, b). Further, although OMM1 and OMM2.5 cells were more sensitive to ABT-263, no additive effect was observed when ABT-263 was combined with siRNA against IRE1α (Supplementary Fig. 4b). siRNA against PERK efficiently inhibited PERK mRNA level as shown by QPCR in both 92.1 and Mel270 cells (Fig. 5a and Supplementary Fig. 5A). Inhibition of total PERK that translated into inhibition of its phosphorylation was also confirmed by western blot analysis in 92.1 cells (Fig. 5b). PERK inhibition also impaired EIF2α activation as revealed by the loss of EIF2α phosphorylation in both basal and ABT-263-treated 92.1 cells (Fig. 5c). To reveal the effect of combined ABT-263 and PERK down-regulation, we decreased the ABT-263 concentration to 3 μM, which did not induce cell death in 92.1 cells (Fig. 1a, b). Whereas ABT-263 treatment or PERK inhibition exhibited a relatively low effect on PARP cleavage and cell death when used alone, the combination significantly increased the effect of ABT-263 and its killing efficacy, illustrated by PARP cleavage and an increase in cell death (Fig. 5b, c). This same holds true for Mel270 cells (Supplementary Fig. 5b, c). We also tested the effect of GSK2606414, a cell-permeable PERK inhibitor. GSK2606414 treatment reduced CHOP and ATF4 accumulation, demonstrating its efficacy in Mel270 (Supplementary Fig. 5d). Whereas GSK2606414 alone displayed no effect on cell viability, the effect of ABT-263 was improved when combined with GSK2606414 (Fig. 5e and Supplementary Fig. 5e), consistent with data obtained with PERK siRNA. Altogether, inhibition of anti-apoptotic BCL-2 proteins by ABT-263 induces a protective feedback response in 92.1 and Mel270 uveal melanoma cells, via induction of the ER stress response that can be prevented with PERK inhibitor.

## Discussion

Metastatic uveal melanoma represents a major clinical challenge since, thus far, there is no efficient systemic treatment. Uveal melanomas are genetically different from cutaneous melanomas and are highly resistant to the therapeutic options used to treat cutaneous melanomas. One of the main mechanisms for cancer cells escaping apoptosis induction is through over-expression or anti-apoptotic proteins, such as the BCL-2-protein family, of which BCL-2 is the prototype.

Certain malignancies, mostly leukemias and lymphomas, appear addicted to a single pro-survival protein (mainly BCL-2). In line with that, BCL-2 transgenic mice exclusively develop hematopoietic malignancies^22^.

BCL-2 was also previously shown to be overexpressed in uveal melanoma compared to normal tissue^23,24^. Previous studies showed that inhibition of BCL-2 alone by antisense oligonucleotides caused cell death of uveal melanoma cells and that reduction in BCL-2 through the use of miR-182 suppressed the in vitro and in vivo growth of uveal melanoma cells^25^. Zeaxanthin, a carotenoid pigment, has also been shown to decrease uveal melanoma cell viability through BCL-2 inhibition^26^.

However, the survival of solid tumors, is often safeguarded by multiple pro-survival BCL-2 family members^10^. Thus, these tumors require drugs targeting both BCL-2 and BCL-xL^10^. Consistent with this notion, our results showed that ABT-199 (mainly directed against BCL-2) lacked efficiency in killing uveal melanoma cells. In contrast, we showed that ABT-263 which targets BCL-2 and BCL-xL has anti-proliferative and pro-apoptotic activities in uveal melanoma cell lines. Noteworthy, in another study, monotherapy with S44563-2, another BCL-2/BCL-xL inhibitor, exhibited a limited effect on uveal melanoma cells; however, the killing efficacy of S44563-2 was improved when combined with fotemustine^24^. Differences in the mechanisms of drug action may explained the discrepancies in ABT-263 and S44563-2 effects. Another explanation might be that the level of BCL-2 and BCL-xL expression varied in the cells selected in these two different studies and thereby influenced their response to ABT-263 and S44563-2.

The BCL-2 protein family has extensively been studied for their role in mitochondrial apoptosis. However, the BCL-2 family seems to play a crucial role also in the endoplasmic reticulum and on the crosstalk with the mitochondria, operating as a stress rheostat^27^. We observed that ABT-263 caused endoplasmic reticulum (ER) stress and activated the unfolded protein response (UPR) in primary melanoma cells, whereas in metastatic cells, ABT-263 elicited only a partial activation of the UPR.

Although BIP increase following ABT-263 treatment was easily seen in Mel270, 92.1 and OMM2.5 uveal melanoma cells, BIP was hardly detected in OMM1 cells. Increased BIP expression reflects the intensity of the UPR activation. Furthermore, CHOP and ATF4 elevation were not detected in OMM1 and OMM2.5 cell lines upon ABT-263 treatment. However, tunicamycin (a known ER stress inducer), enhanced CHOP levels (Supplementary Fig. 3), thereby indicating that this pathway is functional in the metastatic cells and that the lack of ER stress stimulation by ABT-263 is specific.

Thus, most likely, OMM1 and OMM2.5 cells are more sensitive because of partial UPR activation that is not able to counteract the ABT-263 killing effect. It will be interesting to assess whether activation of UPR was employed by uveal melanoma cells used in the previous studies^25,26,28^. In this context, the UPR pathway may be a powerful novel target to improve drug efficacy in the treatment of these uveal melanoma tumors.

Among the three UPR branches, two converge towards IRE1α. Indeed, ATF6 increases *XBP1* mRNA expression while IRE1α mediates its splicing, resulting in the translation of a spliced active form of XBP1 (XBP1s). The PERK-EIF2α axis enhances ATF4. Both XBP1s and ATF4 function as transcription factors that regulate a wide range of genes, which plays a crucial role in cell adaptation to stress conditions^29,30^.

Our results indicate that the protective effect mounted by Mel270 and 92.1 uveal melanoma cells in response to ABT-263 specifically involved the PERK/EIF2α/ATF4 signaling cascade. Indeed, in contrast to IRE1α inhibition that did not change the effect of ABT-263, the combination of ABT-263 with PERK inhibition synergistically reduced the survival rate of primary uveal melanoma cells.

Mel270 and 92.1 which are primary cells appeared more resistant to ABT-263 killing activity than OMM1 and OMM2.5 that are metastatic cells. Interestingly, following ABT-263 treatment, which targets both BCL-2 and BCL-xL, we did not observe in Mel270 and OMM1 cells a compensatory increase in the other anti-apoptotic proteins, ruling out the possibility that a change in the anti-apoptotic protein level causes the different sensitivity of the cell lines to ABT-263.

The difference in sensitivity of primary and metastatic cells may also reflect the addiction of the selected cell lines to pro-survival BCL-2 family members.

Another explanation could be that the uveal melanoma cell lines did not retain the major features of the original tissue. Indeed, we showed that ABT-263 was able to efficiently kill primary uveal melanoma cells that we freshly isolated from a human biopsy (Supplementary Figure 6). We are aware that a higher number of cell lines should be tested to firmly conclude on the response of primary versus metastatic cells to ABT-263 effect. Nevertheless, independently of the tumor stage, we uncovered a resistance mechanism in uveal melanoma cells mediated by activation of endoplasmic reticulum stress pathway. In such context, expression level of ER stress effectors could represent both marker of ABT-263 response and therapeutic targets. Therefore, inhibition of anti-apoptotic BCL-2 proteins by ABT-263 alone or in combination with an ER stress inhibitor represents a potential therapeutic strategy in uveal melanoma treatment.

## Materials and methods

### Cell cultures and reagents

Human uveal melanoma cell lines and short*-*term cultures derived from different patients with metastatic malignant melanoma cells were grown in DMEM supplemented with 7% FBS at 37 °C in a humidified atmosphere containing 5% CO_2_. Lipofectamine RNAiMAX and opti-MEM medium were purchased from Invitrogen (San Diego, CA, USA). ABT-263 was obtained from Euromedex and qVD from Clinisciences.

### Western blot assays

Western blotting was performed as previously described^31,32^. Briefly, cell lysates (30 µg) were separated using SDS-PAGE, transferred onto a PVDF membrane and subsequently exposed to the appropriate antibodies. Antibodies to BCL-2 (ms-123-P0) was from neomarker, to caspase 3 (#610323) was from BD, to BAX (#8429) was from sigma, to PARP (#9542), BCL-XL (#2762), CASPASE 9 (#9502), PUMA (#4976), CHOP (#2895), PERK (#5683), Phospho-PERK (#3179), IRE1α (#3294), BIP (#3177), were from Cell Signaling Technology Inc, to phospho-IRE1α (#NB100-2323) was from Novus, to MCL1 (#sc-819), to NOXA (#sc-56169), and HSP90 (#sc-13119) were from Santa Cruz biotechnology. The proteins were visualized using the ECL system (Amersham). The western blots shown are representative of at least 3 independent experiments.

### Cell death analysis by flow cytometry

Cells were seeded at a density of 100,000 cells/well, in 6-well plate and treated with ABT-263 for indicated time. Cells were harvested using accutase enzyme, washed twice with ice-cold phosphate-buffered saline, resuspended in a buffer (Hepes 250 mM, NaCl 150 mM,KCl 5 mM, MgCl_2_ 2 mM, CaCl_2_ 2 mM) with DAPI (1 µg/ml) and Annexin V- Alexa Fluor 647 conjugate (1/100) and incubated for 15 minutes at room temperature (25 °C) in the dark. Samples were immediately analyzed by a flow cytometer (MACS QUANT) using a laser at 405 nm excitation with a bandpass filter at 425 nm and 475 nm for DAPI detection and a laser at 635 nm excitation with a bandpass filter at 650 nm and 665 nm for Alexa Fluor 647 dye. Annexin and DAPI mono- or double positive cells were counted as dead cells.

### Colony formation assay

Human uveal melanoma cells were seeded onto 6-well plates. The cells were subsequently placed in a 37 °C, 5% CO_2_ incubator. Colonies of cells were grown before being stained with 0.04% crystal violet/2% ethanol in PBS for 30 min. Photographs of the stained colonies were captured. The colony formation assay was performed in duplicate.

### mRNA preparation and real-time/quantitative PCR

The mRNA was isolated using TRIzol (Invitrogen) according to a standard procedure. QRT-PCR was performed using SYBR Green I (Eurogentec, Seraing, Belgium) and Multiscribe Reverse Transcriptase (Applied Biosystems) and subsequently monitored using the ABI Prism 7900 Sequence Detection System (Applied Biosystems, Foster City, CA, USA). The detection of the SB34 gene was used to normalize the results. Spliced Xbp1 primers were previously reported^33^. Primer sequences for each cDNA were designed using either Primer Express Software (Applied Biosystems) or qPrimer depot (http://primerdepot.nci.nih.gov), and these sequences are available upon request.

### Animal experimentation

Animal experiments were performed in accordance with French law and approved by a local institutional ethical committee. The animals were maintained in a temperature-controlled facility (22 °C) on a 12-h light/dark cycle and provided free access to food (standard laboratory chow diet from UAR, Epinay-S/Orge, France). Human Mel270 uveal melanoma cells were subcutaneously inoculated into 8-week-old female, immune-deficient, athymic, nude FOXN1^*nu*^ mice (Harlan Laboratory). When the tumors became palpable (0.1–0.2 cm^3^), the mice received an intraperitoneal injection of ABT-263 (50 mg/kg), dissolved in 10% DMSO 6 times per week. Control mice were injected with DMSO alone. The growth tumor curves were determined after measuring the tumor volume using the equation *V* = (*L* × *W*2)/2. At the end of the experiment, the mice were euthanized by cervical dislocation.

### Statistical analysis

The data are presented as the means + SD and analyzed using a two-way ANOVA or two-sided *t*-test with Graph Pad Prism. The difference between both conditions was statistically significant at *P*-value < 0.05.

## Supplementary information

Supplementary figure legends

Supplementary Figure 1

Supplementary Figure 2

Supplementary Figure 3

Supplementary Figure 4

Supplementary Figure 5

Supplementary Figure 6
