# Supplementary material for: Endoplasmic reticulum stress mediates resistance to BCL-2 inhibitor in uveal melanoma cells
Source: Cell Death Discov. 2020 Apr 17;6:22. doi: 10.1038/s41420-020-0259-2 (PMC7165182; doi:10.1038/s41420-020-0259-2)
Supplement: Supplementary file 1 — Supplementary figure legends [file 41420_2020_259_MOESM1_ESM.docx]

**Supplementary figure legends**

**Supplementary figure 1:** **ABT-263 changes expression of cell cycle effectors.** Mel270 uveal melanoma cells were left untreated or treated with ABT-263 3 μM or 5 μM for 48 h. Representative western blot to CDK4, p27 and p21 are shown. Detection of HSP90 is a loading control.

**Supplementary figure 2: ABT-263 induces mitochondrial membrane potential loss. a-b.** Detection of the mitochondrial membrane potential using TMRE staining in Mel270 and OMM1 uveal melanoma cells exposed to ABT-263 3μM or 5μM for 48h in the absence or presence of qVD-OPh 20 μM. Representative FACS plot of TMRE staining is shown.

**Supplementary figure 3: UPR is expressed in metastatic uveal melanoma cells.** Metastatic uveal melanoma cells were left in control conditions or were treated with ABT-263 3μM or tunicamycin (1μg/ml). Representative western blot to CHOP are shown. Detection of HSP90 serves as a loading control.

**Supplementary figure 4: IRE1**α **knockdown does not impact the ABT-263 effect. a.** Uveal melanoma cells were treated with control (siCtl) or IRE1α (siIRE1α) siRNA for 48 h before being exposed to ABT-263 3μM for 48 h. Representative western blots are shown. Detection of HSP90 serves as a loading control. **b.** Uveal melanoma cells were treated as in (A). FACS analyses of Annexin V/DAPI double staining in uveal melanoma cells indicate alive (white) or dead (early, late apoptosis and necrosis in light grey) cells. *P-value< 0.05**;** ***P-value< 0.001**.**

**Supplementary figure 5: Inhibition of PERK enhances ER killing ability of ABT-263 in Mel270 cells. a.** Q-PCR analysis of PERK level in Mel270 uveal melanoma cells treated with control (siCtl) or PERK (siPERK) siRNA for 48 h. ***P-value< 0.001**. b.** Mel270 uveal melanoma cells treated with control (siCtl) or PERK (siPERK) siRNA for 48 h before being exposed to ABT-263 3 μM for 48 h. Representative western blot to P-EIF2α, total EIF2α and PARP are shown. HSP90 serves as a loading control. **c.** Mel270 cells were treated as in (B). FACS analyses of Annexin V/DAPI double staining in cells freshly isolated from a human biopsy indicate alive (white) or dead (early, late apoptosis and necrosis in light grey) cells. ***P-value< 0.001**. d.** Mel270 uveal melanoma cells were treated with ABT-263 5μM or GSK2606414 5μM alone or in combination for 48h. Representative western blot to CHOP and ATF4 are shown. Detection of HSP90 is a loading control. **e.** FACS analyses of Annexin V/DAPI double staining in Mel270 cells treated as in (D). ***P-value< 0.001**.**

**Supplementary figure 6:** FACS analyses of Annexin V/DAPI double staining in cells freshly isolated from a human biopsy indicate alive (white) or dead (early, late apoptosis and necrosis in light grey) cells exposed to ABT-263 1µM for 48h.
