# Supplementary figures and images for: Endoplasmic reticulum stress mediates resistance to BCL-2 inhibitor in uveal melanoma cells
Source: Cell Death Discov. 2020 Apr 17;6:22. doi: 10.1038/s41420-020-0259-2 (PMC7165182; doi:10.1038/s41420-020-0259-2)

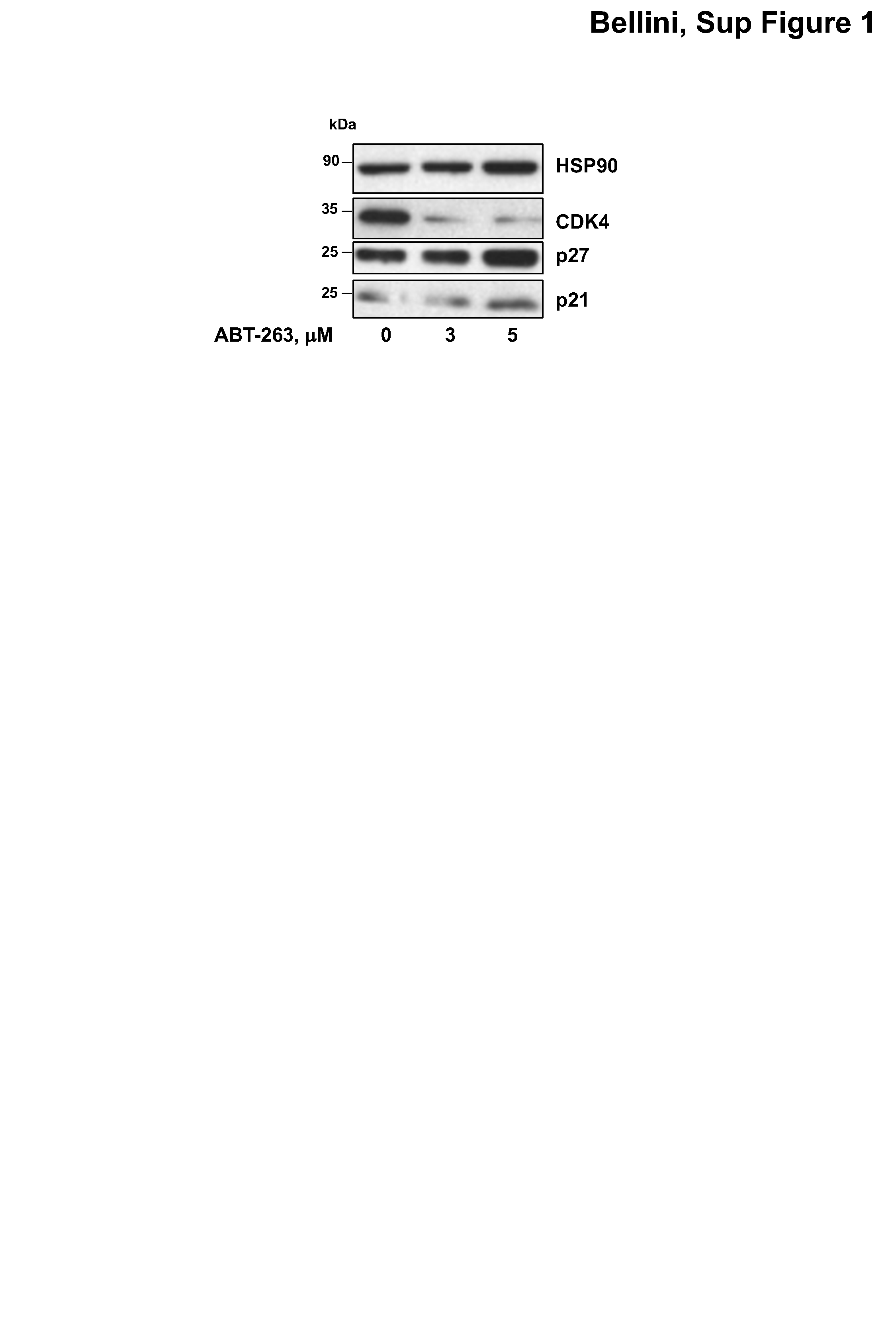

Supplement: Supplementary file 2 — Supplementary Figure 1 [file 41420_2020_259_MOESM2_ESM.tif]

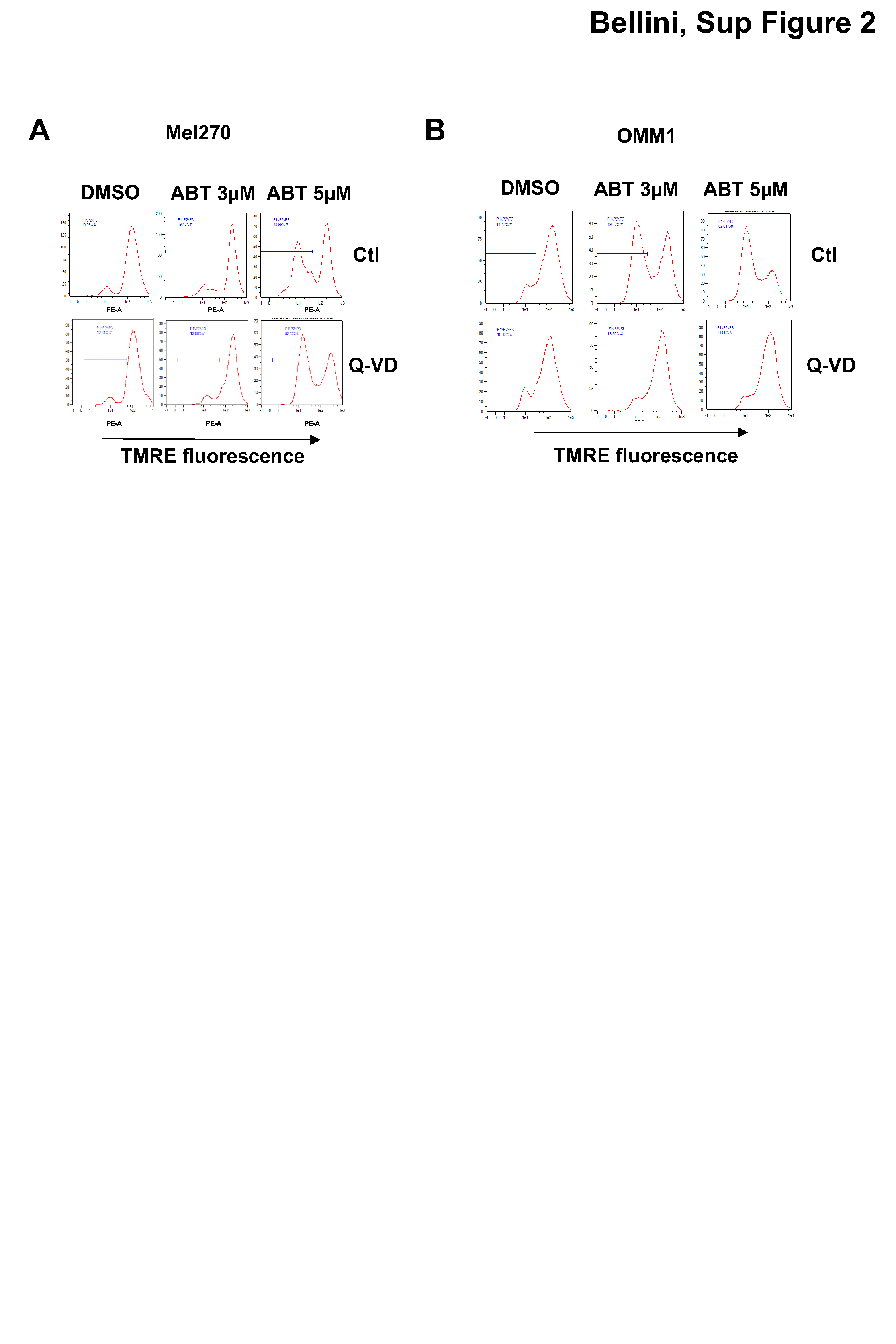

Supplement: Supplementary file 3 — Supplementary Figure 2 [file 41420_2020_259_MOESM3_ESM.tif]

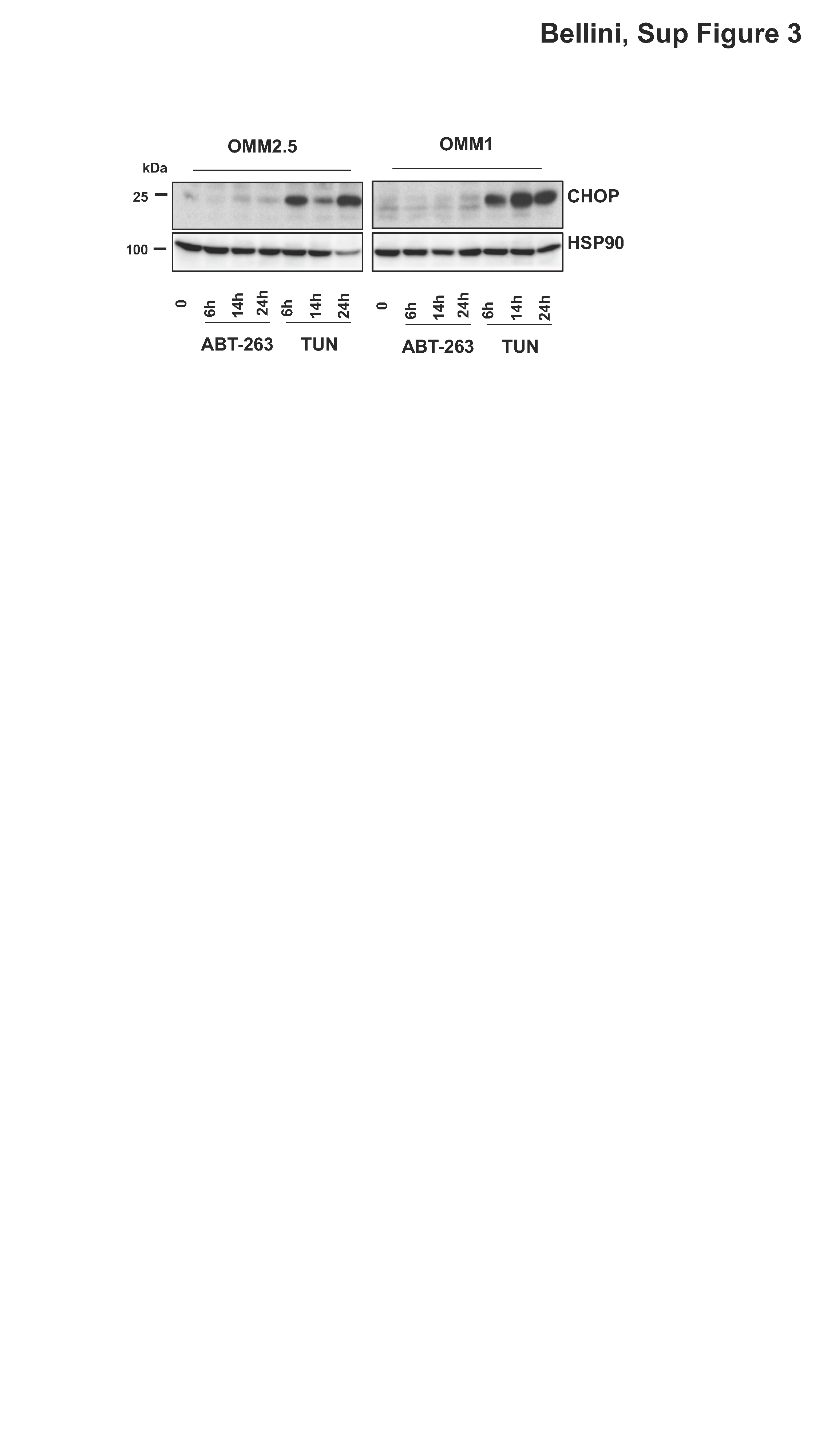

Supplement: Supplementary file 4 — Supplementary Figure 3 [file 41420_2020_259_MOESM4_ESM.tif]

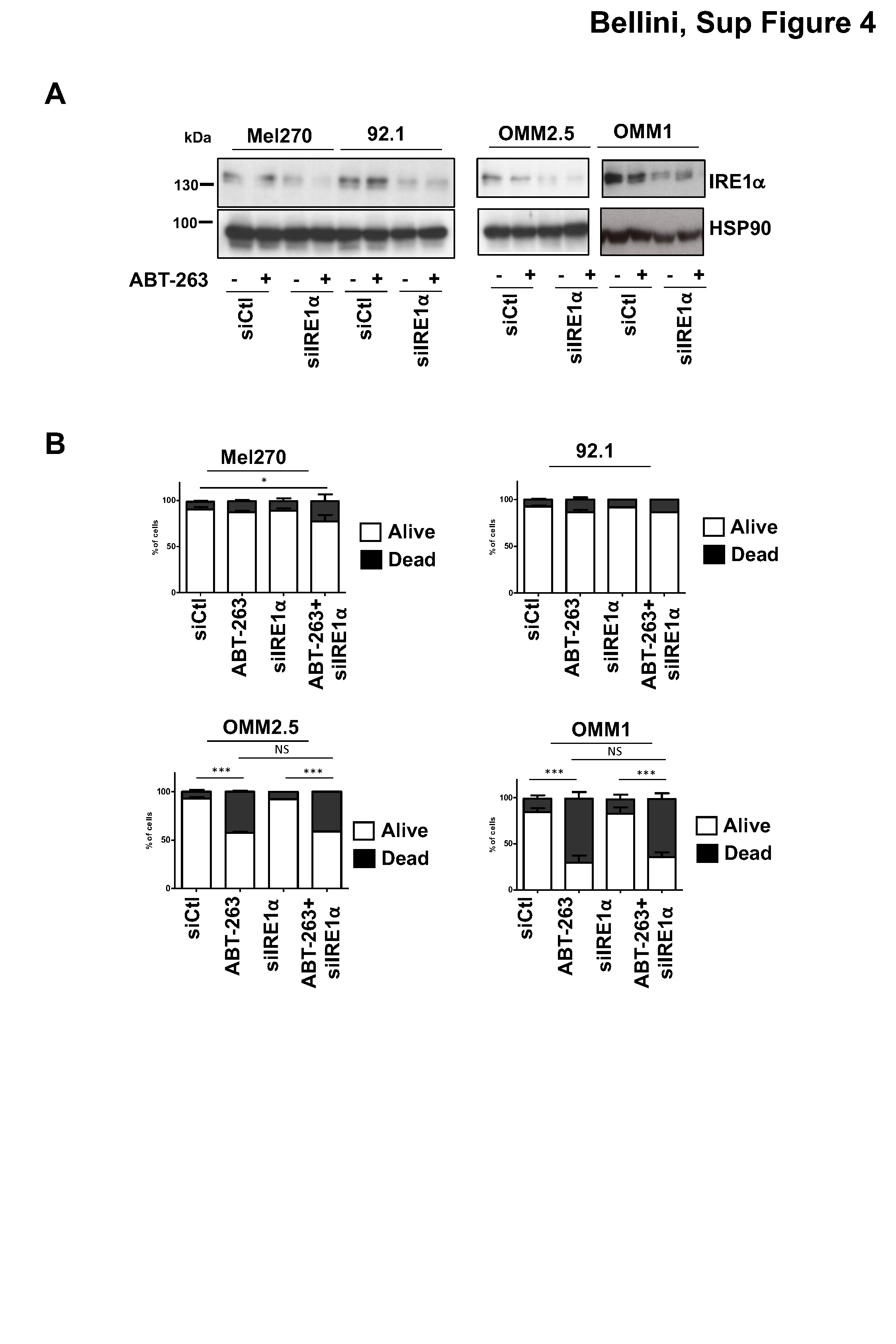

Supplement: Supplementary file 5 — Supplementary Figure 4 [file 41420_2020_259_MOESM5_ESM.tif]

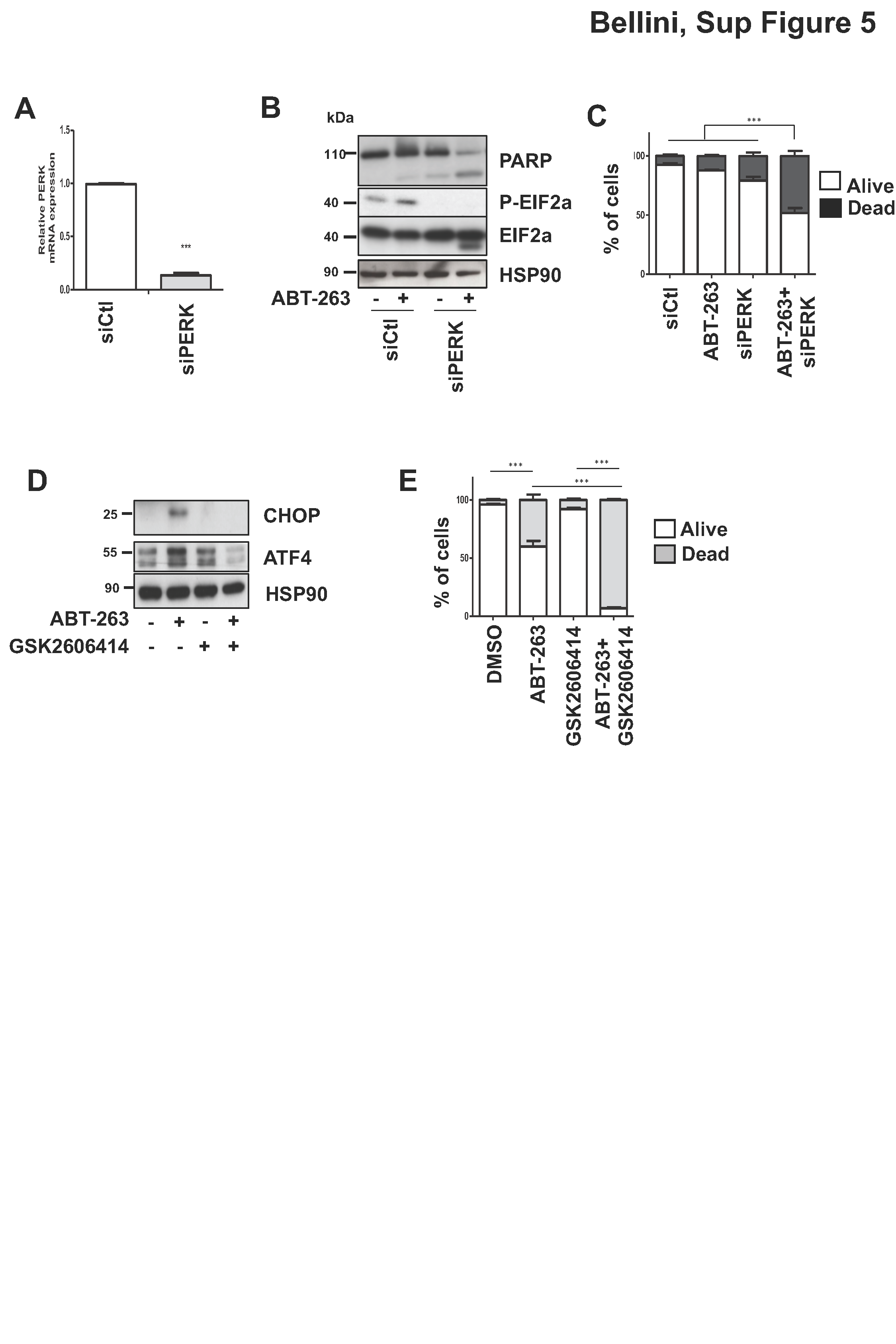

Supplement: Supplementary file 6 — Supplementary Figure 5 [file 41420_2020_259_MOESM6_ESM.tif]
